# Discrimination of contagious and environmental strains of *Streptococcus uberis* in dairy herds by means of mass spectrometry and machine-learning

**DOI:** 10.1038/s41598-018-35867-6

**Published:** 2018-11-30

**Authors:** Necati Esener, Martin J. Green, Richard D. Emes, Benjamin Jowett, Peers L. Davies, Andrew J. Bradley, Tania Dottorini

**Affiliations:** 1 University of Nottingham School of Veterinary Medicine and Science, College Road, Sutton Bonington, Leicestershire LE12 5RD UK; 2 Advanced Data Analysis Centre, University of Nottingham, College Road, Sutton Bonington, Leicestershire LE12 5RD UK; 3 Quality Milk Management Services ltd, Cedar Barn, Easton Hill, Easton, Wells BA5 1DU UK

## Abstract

*Streptococcus uberis* is one of the most common pathogens of clinical mastitis in the dairy industry. Knowledge of pathogen transmission route is essential for the selection of the most suitable intervention. Here we show that spectral profiles acquired from clinical isolates using matrix-assisted laser desorption ionization/time of flight (MALDI-TOF) can be used to implement diagnostic classifiers based on machine learning for the successful discrimination of environmental and contagious *S. uberis* strains. Classifiers dedicated to individual farms achieved up to 97.81% accuracy at cross-validation when using a genetic algorithm, with Cohen’s kappa coefficient of 0.94. This indicates the potential of the proposed methodology to successfully support screening at the herd level. A global classifier developed on merged data from 19 farms achieved 95.88% accuracy at cross-validation (kappa 0.93) and 70.67% accuracy at external validation (kappa 0.34), using data from another 10 farms left as holdout. This indicates that more work is needed to develop a screening solution successful at the population level. Significant MALDI-TOF spectral peaks were extracted from the trained classifiers. The peaks were found to correspond to bacteriocin and ribosomal proteins, suggesting that immunity, growth and competition over nutrients may be correlated to the different transmission routes.

## Introduction

Clinical mastitis is one of the most important challenges facing the dairy industry, where it reduces productivity, profitability and cow welfare. Considerable progress has been made in understanding the epidemiology and microbiology of mastitis over the past four decades, identifying the physical origins of infection as contagious or environmental^1^ and temporal origins of the infection e.g. dry period or lactation^2^. Coliform bacteria are almost always environmental, whilst other pathogens such as *Staphilocossus aureus* and *Streptococcus agalactiae* are typically contagious on the contrary, *S. uberis* can commonly manifest itself in both contagious and environmental forms^3^, and the ability to diagnose the clinical mastitis transmission pattern (contagious or environmental) is an essential step to identify appropriate and effective management interventions for the control of the disease at a herd level. Limited financial and labour resources are typically available to farmers for mastitis control. A role of the clinician is to identify the mode of transmission as early as possible, in order to react appropriately before costly production losses have occurred. Currently, clinicians assess the most likely mode of transmission through analysis of historical data^4^, visual observation of management practices (milking, cleaning, etc.) and knowledge of pathogens, although there is limited evidence that the latter two methods are useful in determining transmission patterns in the modern dairy herd. This leads to inevitable delays and associated losses before a diagnosis of a new, emerging disease pattern can be made. Prompt diagnosis of the likely transmission route in case of an outbreak would allow appropriate control interventions to be implemented earlier and reduce deleterious production and welfare consequences of additional clinical mastitis cases.

Previous studies in this field have used genomic epidemiological techniques to classify individual bacterial strains broadly as contagious or environmental according to their observed patterns of clinical disease within multiple independent herds^4^. These techniques are useful as research tools to understand the observed patterns but are too costly and laborious to be practical clinical tools for clinicians. The discriminatory ability for genomic techniques such as multilocus sequence typing (MLST) may also not be appropriate for the classification of bacterial isolates according to their clinical manifestation if those attributes which govern the transmission behaviour of an isolate are determined by epigenetic factors or conferred by mobile genetic elements which are rapidly exchanged between bacteria such as *S. uberis*^5^.

Evidence of epigenetic strain variation and strain evolution within a bacterial species has been described by several mechanisms, such as differential methylation resulting in phase variation^6^ as a means for isolates of commensal and pathogenic bacterial species to adapt to new or changing environments. In human cases of Salmonellosis, epigenetic strain variation in virulence and host-pathogen interaction could be demonstrated by proteomic analysis where genomic discrimination of strains was not possible^7^. When identifying the route of transmission of an individual bacterial isolate from a case of bovine mastitis, a rapid proteomic technology able to characterise variation in antigenic expression related to virulence, such as surface protein molecules, may be more discriminatory than existing genomic techniques^8^.

Gel-based proteomic techniques for strain differentiation have been used for decades in many species and disease processes including bovine mastitis^9^. More recently, highly discriminatory techniques, such as Matrix Assisted Laser Desorption/Ionization- Time of Flight Mass Spectrometry (MALDI-TOF MS) have increased our ability to investigate the molecular epidemiology and host-pathogen interactions of many bacterial pathogens^10^.

In contrast to genomic techniques, MALDI-TOF MS provides a rapid and economic means of identifying bacteria and has been shown to be capable of strain differentiation within a bacterial species such as *S. pneumoniae, Y. entrocolitica* and *M. pneumoniae*^11–13^.

Comparison of MALDI-TOF MS proteomic profiles may allow discrimination between bacterial, isolate strains of a pathogen, such as environmental *S. uberis* and contagious *S. uberis* strains which have acquired or evolved enhanced survival or colonisation characteristics (genetic or epigenetic) that increase the risk of a cow to cow transmission.

The primary aim of this study was to investigate MALDI-TOF MS data with machine learning as a method to discriminate between *S. uberis* isolates with different modes of transmission; contagious and environmental. A secondary aim of the study was to compare strain differences within and between farms of the UK. The final aim of the study was to identify proteins related to the differentiating peaks between transmission routes with bioinformatics tools.

## Results

### Data source

**Figure 1:**
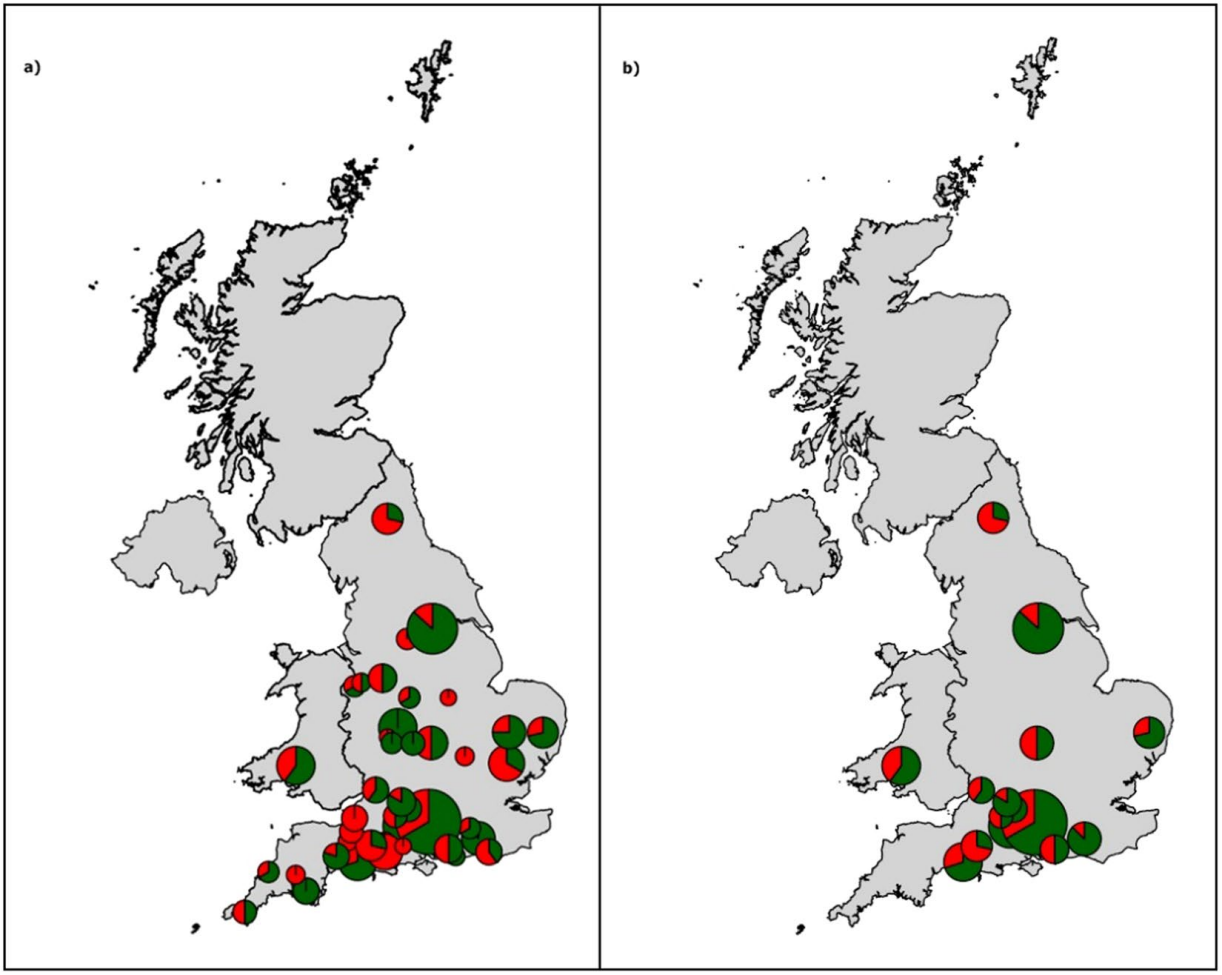
Location of the enrolled farms on the map of the United Kingdom. (**a**) The entire set of 52 farms (**b**) the 19 farms selected for building the model for intra-farm analysis. The red colour represents the environmental isolates of Streptococcus uberis while the green is for contagious ones. The size of the circle indicates the number of Streptococcus uberis isolates in the farms. Figure generated using open source R packages maps and mapdata available from CRAN^66^.

This study used MALDI-TOF spectra and MLST results obtained during a previous study^4^ carried out between March 2004 and May 2005 using selected farms located in England and Wales (see Fig. 1). In that study^4^, *S. uberis* isolates had been cultured from milk samples from 52 herds, each from a different farm (52 farms). The herds were selected only if containing >35 cases of clinical mastitis per 100 cows during a period of 12 months. Within each herd, only cows with a positive diagnosis of mastitis were considered, and each was assigned a clinical classification of either contagious or environmental based on multi-locus sequence typing, as previously described by Davies *et al*.^4^. In such work, the median incidence of clinical mastitis cases had been reported as 66 (mean, 75) per 100 cows per 365 days, with a range of 16 to 146 cases. The mean percentage of clinical cases diagnosed as *S. uberis* had been 28%, with a range in individual herds from 7% to 64%.

In this work, we first assessed the geographical distribution of clinical cases. The results are shown in Fig. 1, indicating spread in England and Wales, with a higher concentration towards the south, and no herds in Scotland.

**Figure 2:**
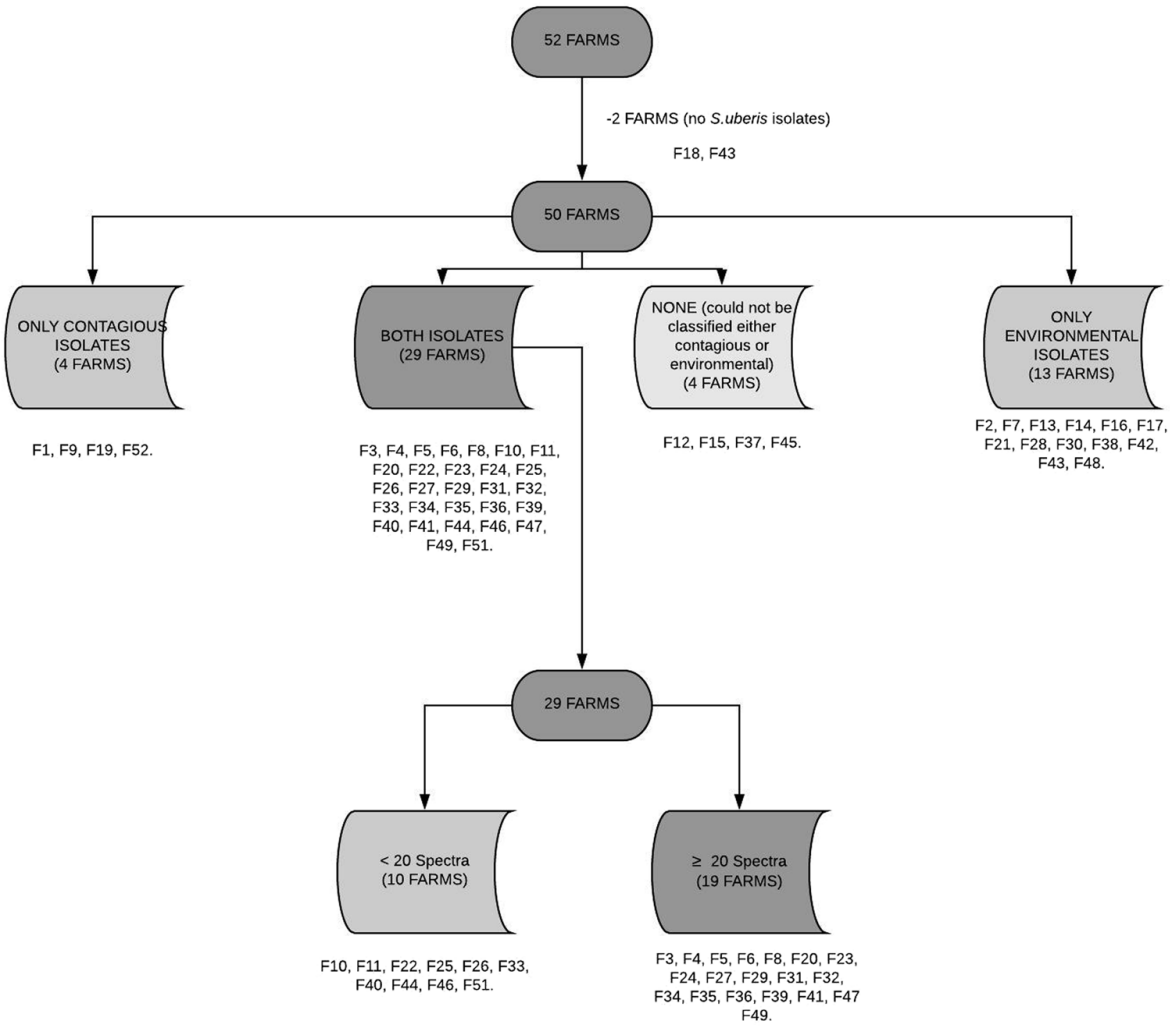
Process of initial farm selection and farm codes. Categorisation was done according to type and presence of Streptococcus uberis (contagious and environmental) strains and number of MALDI-TOF spectra.

In order to construct predictors/classifiers, we focused on herds containing both environmental and contagious *S. uberis* isolates. Thus, by looking at the available MLST data, we selected for the study only the 29 farms/herds containing both. The 23 eliminated herds consisted of: 2 not containing any *S. uberis* isolates, 4 containing only contagious, 13 containing only environmental and 4 containing unclassified isolates (see Fig. 2). Amongst the 29 herds selected, 10 were reserved for external validation (holdout group) as they each featured <20 MALDI-TOF spectra, too few to be useful for the generation of effective classifiers. The remaining 19 farms (Fig. 1b) were considered suitable for the development of classification models.

### Generation of MALDI-TOF peak lists and set-up of the classifiers

The original MALDI-TOF raw spectra had been obtained in the previous study using Bruker technology^3^. In this work, peak lists, i.e. lists of paired mass/charge (m/z) ratios and corresponding intensity values, were extracted from the raw spectra as specified in the Methods section.

To verify if MALDI-TOF peak lists associated with isolates could be used to predict their contagious or environmental nature, supervised machine learning technologies were used to implement classifiers, i.e. software systems that, once provided with a spectrum as the input, would respond by predicting the most likely class (i.e. environmental or contagious) for the isolate. Being based on supervised learning, all methods required the availability of training datasets for model building^14^ (i.e. peak lists with known associated classification of environmental or contagious from MLST), and validation datasets for assessing the performance of the classifier.

### Classification methods

The following classification methods available in the Bruker mass spectrometry analysis software ClinProTools 3.0^15^ were tested: Genetic Algorithm (GA), Supervised Neural Network (SNN) and Quick Classifier (QC).

The GA method uses the training datasets to identify a subset of peaks shared by all of the peak lists (referred to as “peak combination”), acting as the most effective discriminator between contagious and environmental isolates. The performance of each tentative combination in terms of discrimination effectiveness is assessed by evaluating the degree of separation of the clusters formed by the known contagious and environmental isolates, once that combination of peaks is considered. The identification of the best combination is treated as an optimisation problem and solved via a genetic algorithm^16^. When the best peak combination is found (end of training), new peak lists submitted to the classifier are predicted as being contagious or environmental by extracting the peak combination, and using it to determine which one of the two clusters is closest to the observation using the k-nearest neighbour (KNN) metric^15^.

The SNN method is based on building a classifier powered by a neural network implementing a modified version of the supervised relevance neural gas algorithm^17^. The peak lists of the training set are investigated by the algorithm, to identify “prototype” lists suitable to act as representative for the corresponding class (contagious or environmental). Once trained, the network can be fed with any new peak list; the prediction will be based on understanding which prototypes are closer to the given list^15^.

The QC method is based on grouping the peak lists into two classes (environmental and contagious), generating peak averages representative of each class, and ranking the relevance of the peaks when acting as discriminators based on statistical testing^15^. Any new observation is then tested on similarity against the weighted averages of each class, the most similar class being elected as the prediction result^15^.

### Prediction performance

The prediction performance of each classifier was evaluated by considering the following indicators, assuming P and N as the total number of positive (contagious) and negative (environmental) MLST results, and using T to indicate true (correct) and F to indicate false (wrong) predictions:

- sensitivity (TPR - true positive rate) = TP/P
- specificity (TNR - true negative rate) = TN/N
- precision (PPV - positive predictive value) = TP/(TP + FP)
- NPV - negative predictive value = TN/(TN + FN)
- accuracy (ACC) = (TP + TN)/(P + N)
- Cohen’s kappa statistics = (*p*_*o*_ − *p*_*e*_)/(1 − *p*_*e*_)

where:

1 $${p}_{o}=({\rm{T}}{\rm{P}}+{\rm{T}}{\rm{N}})/({\rm{P}}+{\rm{N}})\,{\rm{a}}{\rm{n}}{\rm{d}}\,{{\rm{p}}}_{{\rm{e}}}=({\rm{P}}\cdot ({\rm{T}}{\rm{P}}+{\rm{F}}{\rm{N}})+{\rm{N}}\cdot ({\rm{F}}{\rm{P}}+{\rm{T}}{\rm{N}}))/{({\rm{P}}+{\rm{N}})}^{2}$$

Based on the above indicators, the following performance parameters were considered:

- recognition capability (RC): the accuracy obtained when the classifier is trained with the entirety of the dataset and tested on the same data.
- accuracy, kappa and other indicators from cross validation (CV): the dataset is split in a training subset (containing a percentage of the available spectra) and a testing subset. (containing the remaining spectra). The subsets are created by randomly drawing individuals from the same dataset. Accuracy, kappa and any other desired indicator resulting from the confusion matrix are computed. The entire process is repeated n times (training and testing n classifiers), then the final indicators are obtained as the arithmetic means of the values obtained on the n confusion matrices.
- accuracy, kappa and other indicators from external validation (EV): only one classifier is built, by using the entirety of the available dataset for training. Testing is performed using a dataset separate from the training dataset. The indicators are computed on the resulting confusion matrix.

### Intra-farm analysis

In intra-farm analysis, classifiers were developed to operate exclusively within each individual farm. The spectra of the 19 farms selected as suitable for model building (Fig. 2) were used to implement 19 separate classifiers (one per farm). Only data pertaining the specific farm were used to implement and validate each classifier. RC and CV performance indicators were computed for each farm, but no external validation was performed.

**Figure 3:**
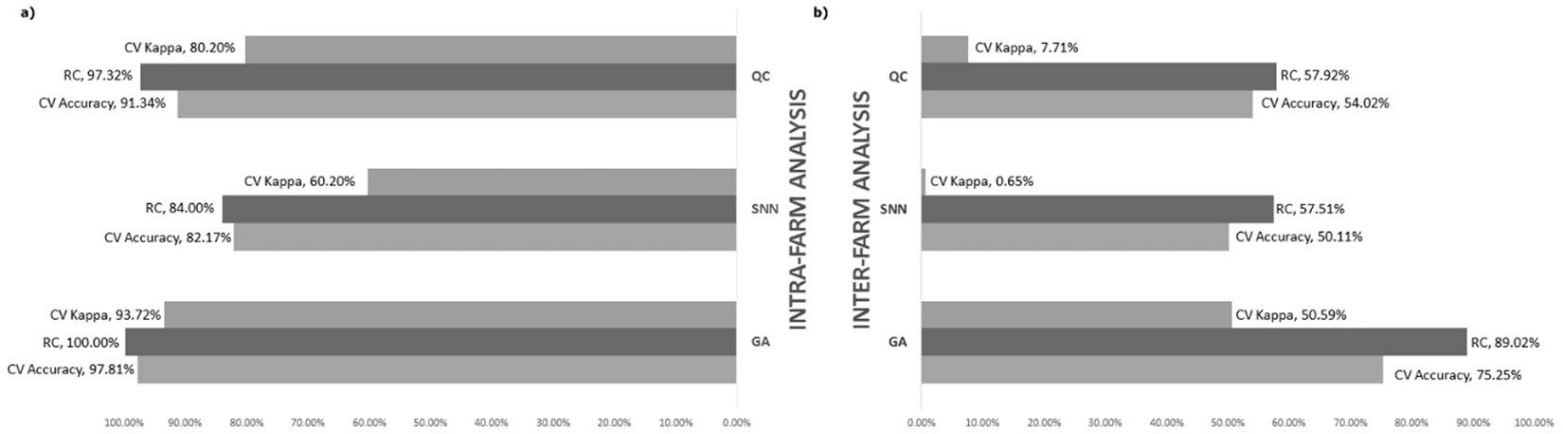
Comparison of intra-farm (**a**) and inter-farm (**b**) analysis results of 19 farms using Genetic Algorithm (GA), Supervised Neural Network (SNN) and Quick Classifier (QC). Inter-farm analysis results are the arithmetic mean of the results from nineteen classifiers (one per farm). All the results were obtained by adopting the default settings for the classifying methods.

The results of the intra-farm analysis on the 19 selected farms are shown in Fig. 3a. Classifiers were run with default settings as described in the Methods section. Recognition capability (RC), cross validation (CV) accuracy and kappa are shown as arithmetic means computed from the individual results of the 19 farms (GA: RC = 100.00%, CV accuracy = 97.81%; CV kappa = 93.72%, sensitivity = 97.13%, specificity = 96.26%, PPV = 98.03%, NPV = 96.19%. SNN: RC = 84.00%, CV accuracy = 82.17%, CV kappa = 60.20%, sensitivity = 87.22%, specificity = 72.65%, PPV = 80.09%, NPV = 84.25%. QC: RC = 97.32%, CV accuracy = 91.34%, CV kappa = 80.20%, sensitivity = 92.36%, specificity = 87.20%, PPV = 91.60%, NPV = 90.33%).

### Inter-farm analysis

In inter-farm analysis, a single classifier was developed and trained on the aggregated data from all the available 19 farms. RC, and performance indicators from cross validation were computed using the data from the 19 farms. The RC and CV results for the inter-farm analysis are shown in Fig. 3b. Classifiers were run with default settings (GA: RC = 89.02%, CV accuracy = 75.25% CV kappa = 50.59%, sensitivity = 81.75%, specificity = 69.16%, PPV = 82.98%, NPV = 67.32%. SNN: RC = 57.51%, CV accuracy = 50.11%, CV kappa = 0. 65%, sensitivity = 64.09%, specificity = 36.55%, PPV = 57.98%, NPV = 42.70%. QC: RC = 57.92%, CV accuracy = 54.02%, CV kappa = 7.71%, sensitivity = 66.97%, specificity = 40.59%, PPV = 61.19%, NPV = 46.77%). The GA method was selected for further optimisation, due to better performance. With the optimised settings (see Methods), the performance of the GA classifier went from 89.02% to 99.09% for RC, from 75.25% to 95.88% for CV accuracy, and from 50.59% to 92.62% for CV kappa.

External validation was performed on the GA classifier, using the additional 10 farms in the holdout group. The following EV indicators were obtained: sensitivity 82.07%, specificity 50.00%, PPV 74.84%, NPV 60.73%, accuracy 70.67% and Cohen’s kappa coefficient 33.80%.

**Figure 4:**
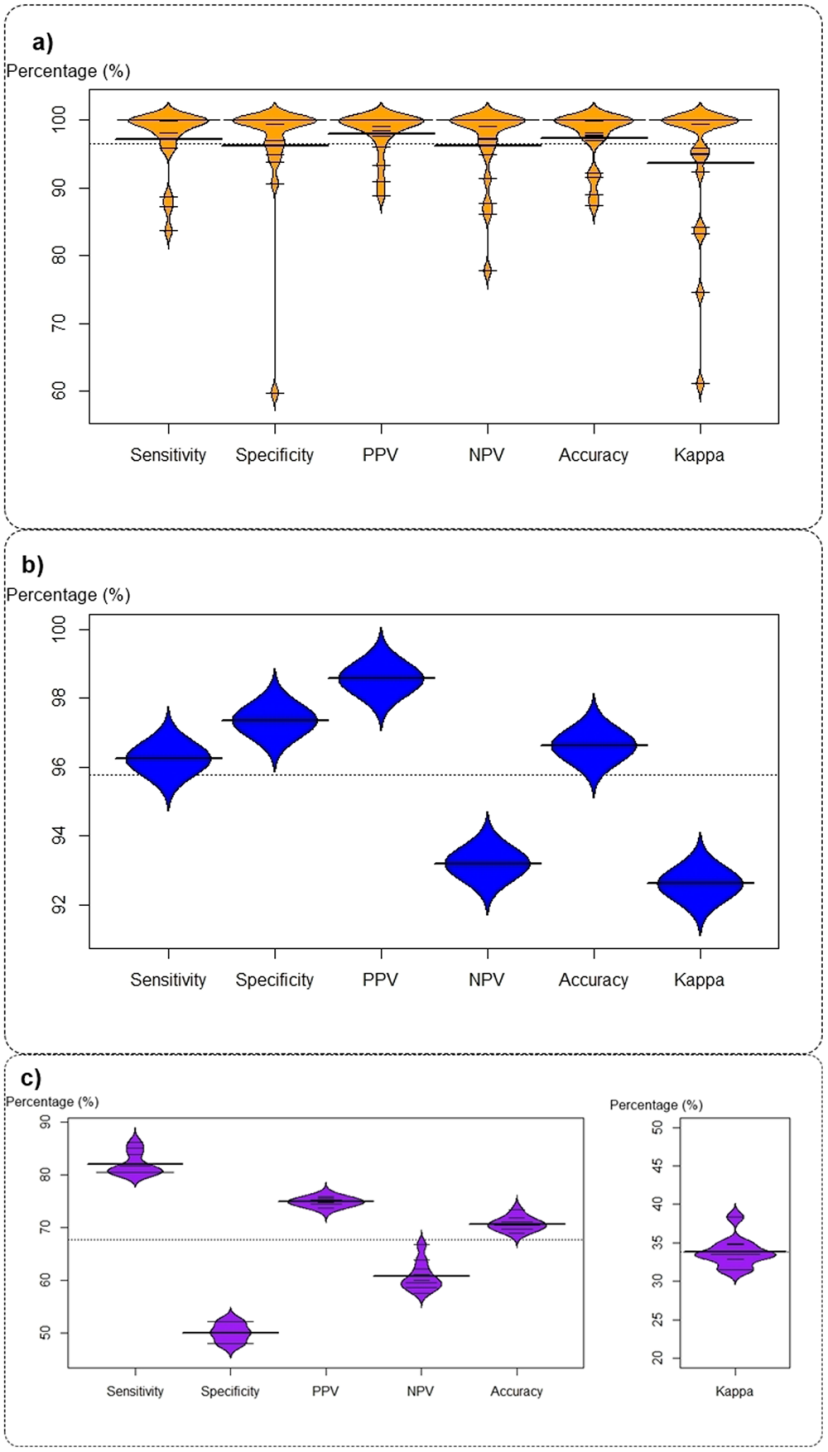
Distribution of the performance indicators for the classifiers/predictors. (**a**) intra-farm cross-validation; (**b**) inter-farm cross-validation; (**c**) inter-farm external validation. Data from 19 farms.

The probability distributions of the performance indicators for intra-farm and inter-farm analysis are reported in Fig. 4.

### Biomarker characterisation

The GA classifier for the intra-farm analysis identified a set of 19 peaks shared by all the isolates, providing optimal discrimination between environmental and contagious isolates.

The masses of these peaks were then compared with the molecular weights of *S. uberis* proteins in NCBI database and 7 out of 19 could be matched to 8 proteins from the proteome (in one case, the molecular weight of a peak was close to two different proteins). The entire procedure is described in the Methods section.

**Figure 5:**
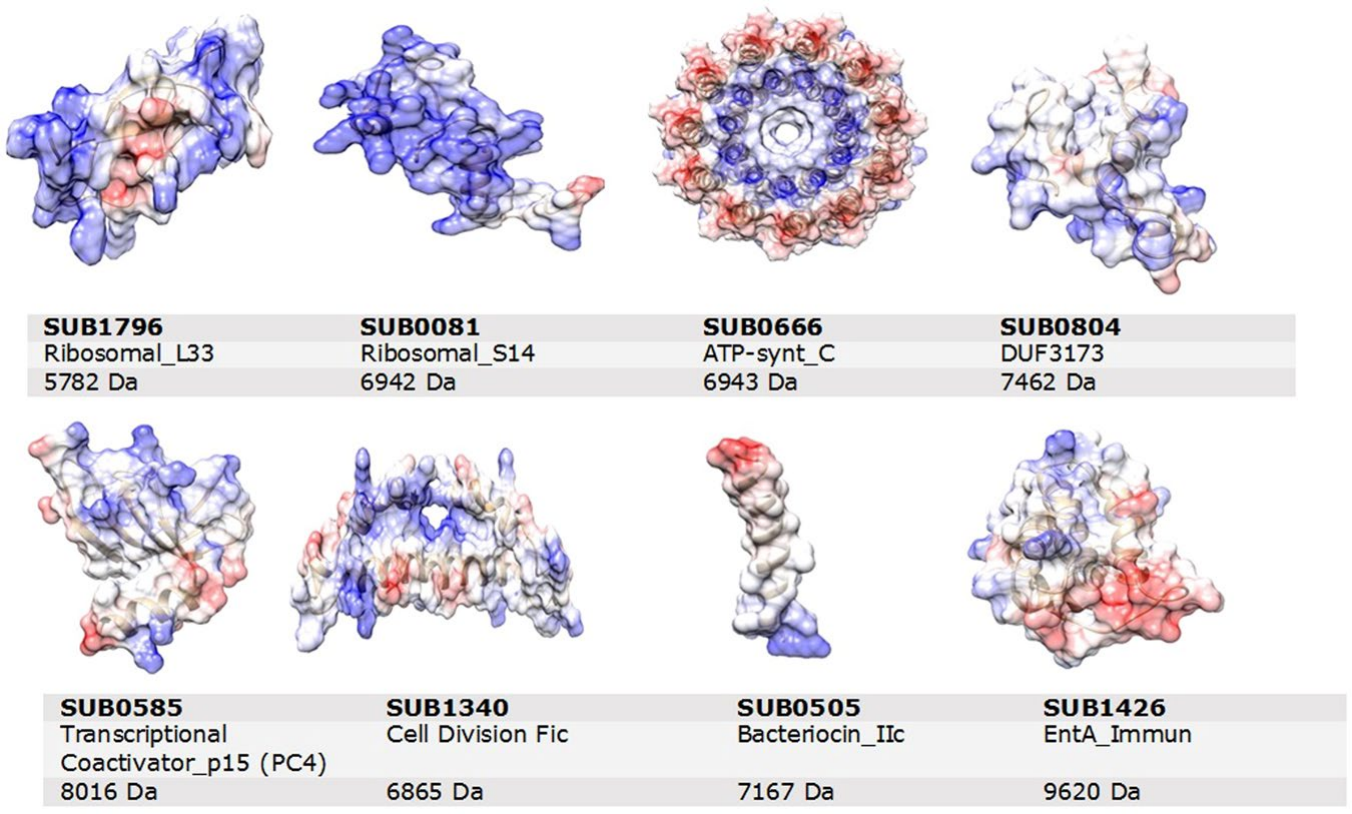
Selected proteins of Streptococcus uberis. Top to bottom: 3D protein structure, Protein ID, Domain of the protein and Molecular weight of the protein.

Five of the eight proteins have a known function (according to NCBI): two of them are ribosomal proteins, two of them bacteriocins and one is an ATP synthase protein. The remaining three proteins had unknown functions; two of these were hypothetical and one of them was of an unknown domain. Using the SMART database, the domains of the 8 proteins were found. The predicted three-dimensional models are shown in Fig. 5.

**Figure 6:**
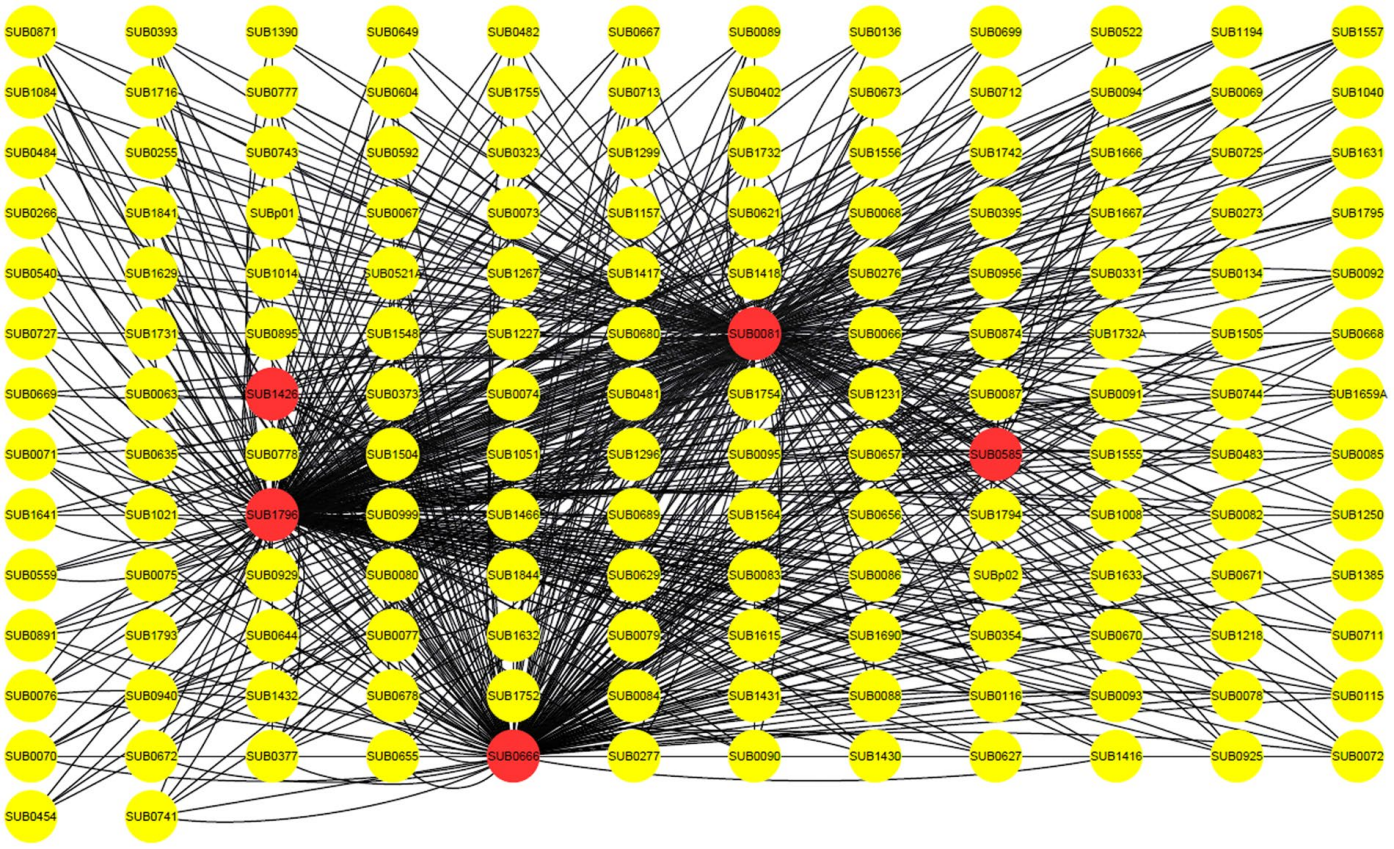
The protein-protein interaction (PPI) network showing 153 Streptococcus uberis proteins (yellow) interacting with the 5 discriminant proteins (red).

**Figure 7:**
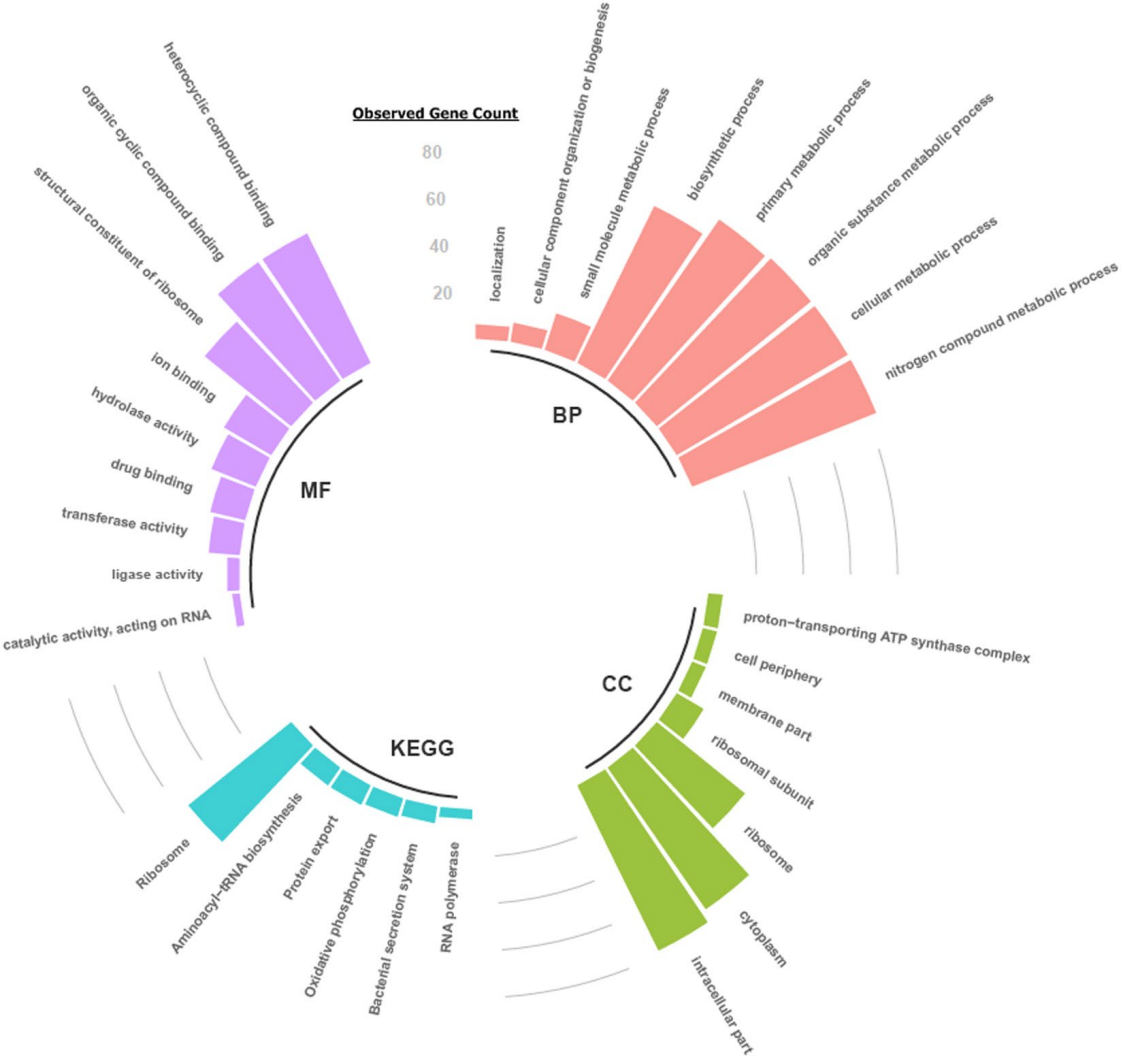
Functional annotation of 158 proteins (5 of interest and 153 interacting with at least two genes of interest) in Streptococcus uberis, based on Gene Ontology and KEGG Pathway.

The analysis of the protein-protein interaction (PPI) network for the discriminant proteins in *S. uberis* (Fig. 6) showed that 5 out of 8 proteins (SUB14226, SUB0666, SUB0081, SUB1796 and SUB0585) share common first neighbour proteins with each other. Interestingly, SUB0666, SUB0081 and SUB1796 were also found to interact with each other. The ontology functions of these 158 proteins (5 of interest and 153 connected to at least two genes of interest) are shown in Fig. 7 as well as KEGG pathways they are involved in (FDR < 0.05).

## Discussion

Our study shows that *S. uberis* isolates classified according to transmission route as either contagious or environmental can be discriminated by MALDI-TOF spectral profiles. The discriminatory power of MALDI-TOF appears to be greater in intra-farm analysis, in particular when using genetic algorithms, indicating the potential for the development of successful screening solutions at the herd level. Inter-farm classification does not work equally well, indicating that more work is needed to develop screening solutions applicable at the population level. Such limited classification performance may be due to divergent evolution of bacterial strains across farms. Contact between dairy farms within the UK is limited, and management practices to control environmental mastitis vary (e.g. different choices of bedding materials, different cleaning practices, different antibiotic treatment protocols). Thus, divergent evolution and hence the emergence of farm specific populations of *S. uberis* is entirely plausible. Diversity within common bacterial species has been demonstrated, such as for *Campylobacter* populations between broiler farms in Switzerland^18^, and *Staphylococcus aureus* strains in bovines from different geographical regions in Argentina^19^. At a much larger scale, divergent evolution of *Helicobacter pylori* has been described in human populations migrated from a common origin to different geographical regions^20^. Because of such diversity, it is possible that the same combination of discriminant peaks may not work equally well for all the farms.

For the investigated data, classifiers based on the GA method showed better performance over SNN and QC both before and after being optimised.

The results showed the presence of proteomic phenotypic differences between contagious and environmental strains of *S. uberis* along with previously demonstrated, different genotypic characteristics^4^. This would appear plausible since selection pressure in the mammary glands is likely to force changes in protein expressions.

In this study, seven of the 19 peaks identified by the GA as the most discriminant between environmental and contagious isolates, where found corresponding to the protein products of eight genes in the reference 0140 J genome. While the GA is treating the identification as a purely mathematical optimization problem, it is interesting to see if the mapped proteins have some functional meaning that might explain their differentiating power. This can be done by looking at gene ontology (GO), where protein functions are annotated according to the aspects of biological process, molecular function and cellular component (see Methods).

The SUB1796 protein is the ribosomal protein-L33 (RP-L33), a relatively small protein consisting of 49 to 66 amino acids, part of the large ribosomal subunit^21^. In GO, RP-L33 is annotated as follows: biological process – translation; molecular function – structural of constituent of ribosome; cellular component – intracellular and ribosome. RP-L33 was shown to have paralogs differing on whether it binds structural zinc or not, which helps bacteria survive in the case of zinc starvation^22^. This is an important consideration, as most of the ribosomal proteins are encoded by single and highly conserved genes. Zinc has a crucial role in preserving the immune status of the cow and mammary gland health; its deficiency can result in an increased mastitis incidence^23^. RP-L33 was discovered to be phylogenetically distinct amongst bacterial species and within single genomes such as that of *Bacillus subtilis*, *Lactococcus lactis*, *Mycoplasma pneumonia*, *Mycoplasma genitalium*, *Ureaplasma ueralyticum* and *Streptomyces coelicolor*^24^. Moreover, RP-L33 was demonstrated to be a core member of the minimal set of bacterial genes which is essential to maintain cell life^25^. RP-L33 was suggested as the putative drug target in three *Streptococcus* species (*S. agalactiae*, *S. pneumoniae*, and *S. pyogenes*)^26^, two *Bordetella* species (*B. pertussis* and *B. parapertussis*)^27^, *Helicobacter pylori*^28^ and *Mycobacterium tuberculosis*^29^.

The SUB0081 protein is the ribosomal protein S-14 (RpsN). The GO annotation is: biological process – translation; molecular function – metal ion binding, rRNA binding, and structural constituent of the ribosome; cellular component - ribosome. RpsN was shown to be essential for growth and deficiency of RpsN resulted in incomplete 30S subunits in *Bacillus subtilis*^30,31^, *E. coli*^32^ and *Staphylococcus aereus*^33^.

The SUB0666 protein is an ATP synthase (subunit C, atpE). The GO annotation is: biological process - ATP hydrolysis and synthesis, and proton transport; molecular function - hydrogen ion transmembrane transporter, and lipid binding; cellular component – plasma membrane. The atpE gene was targeted by several drug studies such as R207910^34,35^ and Bedaquilline^36^ in mycobacterium species. Moreover, the C sub-unit was shown to be the target site of venturicidin in several *E. coli* studies^37,38^.

The SUB0585 protein is the transcriptional coactivator p15 (PC4). The GO annotation is: biological process - regulation of transcription, DNA-templated; molecular function - DNA binding and transcription coactivator; cellular component – no terms assigned. PC4 is also believed to have an important role in DNA repair of bacterial species since studies with *E. coli*^39^ and *Leptospira* species^40^ revealed that PC4 protected the DNA during oxidative stress.

The SUB1340 protein (GO annotation: no terms assigned) contains the Fic domain. This domain is often found in pathogenic and non-pathogenic bacteria with different structures, where some families may contain conserved regulatory functions^41^. It is suggested that pathogenic bacteria secrete Fic proteins, thus showing similar functionality to toxins in terms of fulfilling some duties in the host cell such as interfering with cytoskeletal, signalling and translation pathways^41^. Fic proteins participate to cell division and have been shown to synthesize folate in *E. coli*^42^. In turn, folate is involved in the secretion of enzymes controlling the bacterial pathogenesis in cattle pathogenic bacteria, *Histophilus somni*^43^.

The SUB0505 protein is a bacteriocin. It has the following GO annotation: biological process - defense, response to bacterium; molecular function – no terms assigned; cellular component – no terms assigned. Many bacteria produce tiny peptides called bacteriocins for antimicrobial activity to compete with intra and interspecies over limited nutrients in the environment^44^. In the study by Ward, *et al*.^45^, six bacteriocin proteins including SUB0505 were found in the 0140 J strain, where this redundancy was interpreted as the result of mutations. The study done by Hossain, *et al*.^46^ revealed the absence of bacteriocin genes, including SUB0505, in the EF20 strain, which may be correlated to the non-virulent status of the EF20 strains. The EF20 strain of *S. uberis* was shown to be susceptible to phagocytosis by bovine neutrophils in the presence of serum^47^, and mammary gland macrophages were reported to have capability of killing the EF20 strain in the media containing 50% skimmed milk as the source of opsonin or 10% pooled bovine serum^48^. Moreover, a comparison of the EF20 strain with the reference strain 0140 J showed EF20 growing relatively slowly in raw skimmed milk^49^. In another study^50^, EF20 strains of *S. uberis* performed a high amount of bounding plasmin activity following growth while bovine plasminogen was present in the media. Bacteriocin immunity proteins prevent the bacteria from the toxic effect of its own bacteriocins by forming a stable compound with the receptors^51,52^. The EntA immune protein SUB1426 (no GO annotation) was discovered to guard the particular bacteria against its own class II bacteriocins^53^. The studies in *Streptococcus* species revealed that immunity proteins play a significant role in antimicrobial sensitivity by regulating quorum-sensing^54,55^. In summary, the literature shows that bacteriocins feature high levels of differentiation depending on environment and host immunity response. This may justify why we found SUB0505 as a discriminating peak between environmental and contagious strains of *S. uberis*.

Interestingly, the protein network analysis showed that three of the identified proteins (SUB0081, SUB0666 and SUB1796) interact with one another. This may suggest that the functions (ribosome, oxidative phosphorylation and bacterial secretion system) and importantly the expressions of key proteins participating in the differentiation of transmission routes of *S. uberis*, are co-ordinated. The co-ordination implies that the regulatory changes acting on these genes are accumulated over time across strains. Such stringency in constraining expression variance shifts may play an evolutionary role in the definition of different phenotypically-related traits.

The results of this study suggest that MALDI-TOF spectral analysis of clinical mastitis isolates could provide a rapid means of diagnosing the likely route of transmission at the early stages of a mastitis outbreak, as previously suggested by Archer, *et al*.^56^. Given that potentially contagious transmission of *S. uberis* has been identified in two-thirds of commercial herds and it has been found as the dominant transmission route in a third of UK herds^56^, there is a clear need for diagnostic tools capable to discriminate between contagious and environmentally acquired infections. Tools based on MALDI-TOF spectral analysis would enable clinicians to identify the most appropriate control measures promptly, during an outbreak of disease. A diagnosis based on MALDI-TOF spectra has the potential to reduce the incidence of clinical disease, reduce associated production losses, reduce the costs associated with treatment of clinical mastitis and improve the efficiency of labour and resource allocation on farm.

In conclusion, the analysis of MALDI-TOF spectral profiles through solutions powered by machine learning, and in particular genetic algorithms, was shown to be useful to predict the contagious or environmental nature of *S. uberis* mastitis. Classifiers developed to target individual farms achieved 97.81% CV accuracy (mean over 19 farms), with a mean Cohen’s kappa coefficient of 94%, clearly indicating the possibility to deploy effective diagnostic solutions capable to distinguish between environmental and contagious *S. uberis* strains within a farm. Prediction performance was still high at cross-validation for a classifier trained and tested on the aggregation of the data available from the same 19 farms (CV accuracy 95.88%, kappa 92.62%) but dropped to accuracy 70.67% (kappa 33.80%) when the predictor was externally validated with data from ten additional farms left as holdout. It is unclear at the moment if such degradation may be due inherent proteomic diversity in *S. uberis* populations between herds, and if performance may be improved by simply increasing the amount of data available to train the predictors, for example by including a larger number of farms, or by focusing on ensuring more variation within the training sets. In any case, elucidating the role of specific proteins that have been found discriminatory between contagious and environmental transmission, may provide insights into the underpinning biology of the pathogen. The protein network analysis has also shown the presence of a protein functional network suggesting the existence of a constrained co-evolution of functional pathways and protein expression in participating in differentiating transmission routes of *S. uberis*.

As future endeavour, it may be interesting to investigate whether similar solutions based on the analysis of MALDI-TOF spectra by means of machine learning may be used to develop screening tools to identify early signs of mastitis, or related risk factors. Such a research goal has not been covered yet by our studies, as a significantly different approach is required both in terms of planning and executing data collection and for validating the results. Nevertheless, the analysis of MALDI-TOF peaks has proven successful at discriminating between contagious and environmental strains of infected animals, in particular within individual herds, and one may wonder if a discrimination between healthy and infected individuals may be carried out by looking at similar sets of peaks.

## Methods

### MLST and MALDI-TOF datasets

In order to better appreciate the nature of the available data, the following information is provided from previous work^4^.

MLST: for the classification of environmental and contagious *S. uberis* strains, samples were cultured by a commercial milk laboratory. Putative *S. uberis* colonies were stored on beads at −80 °C. The gDNA was extracted for MLST sequencing in accordance with the protocol described in previous literature^57^. Clinical cases attributed to isolates of the same multi-locus sequence type (MLST) occurring in different cows in the same herd within a 42 day time period were classified as contagious, whereas cases attributed to isolates occurring only once in any cow of any herd were classified as environmental.

MALDI-TOF spectra: all isolates classified as contagious or environmental were incubated on blood agar at 37 °C for 18 to 24 hours. Protein extraction was conducted as previously described^58^ by means of a Time-of-flight (TOF) MALDI mass spectrometer (Bruker Daltonics, Billerica, MA). For each *S. uberis* isolate, 6 technical replicate profiles were generated from 40 desorptions per replicate. Spectra were compared visually using Biotyper 3.1 (Bruker Daltonics). Those with insufficient resolution, low intensity, or substantial background noise were removed. Technical replicates were further compared using composite correlation indices (CCI) to remove dissimilar spectra with CCI < 0.99^59^.

In this work, to extract each peak list, the following steps were applied in ClinProTools 3.0^15^:

- baseline subtraction: using the Top Hat baseline (minimal baseline width: 10%)^60^;
- normalisation: to the total ion count, leading to spectral intensities in the [0–1] range^15^;
- recalibration of the m/z values: using as reference masses those appearing in at least 30% of the spectra and setting 1000 ppm as the maximal peak shift^15^;
- total average spectrum calculation: using weighted contributions from the available replicates^15,61^;
- average peak list calculation: the calculation was applied on the total average spectrum rather than on each single spectrum^15^;
- peak picking on the total average spectrum: using resolution: 800 for spectrum smoothing, signal to noise threshold: 5.00, and 0.000% relative threshold base peak to include all peaks^15^;
- peak normalization: to give the same relevance (weight) to all peaks within the classification/prediction models^15^;
- mass range filter: the mass range of the spectra was limited to 4–10 KDa.

### Parameters used for the classification methods

The GA default parameters (as suggested by the ClinProTools software) were as follows: initial number of peak combinations (INPC): automatic detection; maximal number of peaks (MNP, maximum number of peaks to be included in the combination): 5; maximum number of generations (MNG, number of GA iterations to identify the optimal result): 50; mutation rate (MR): 0.2; crossover rate (CR): 0.5 (mutation and crossover control how new candidate combinations are created starting from those tested in the current generation); number of neighbours for KNN (KNN, number of neighbours considered by the KNN method to determine the distance of a new observation from an existing class): 50. The optimised set of GA parameter was: INPC: 125, MNP: 19, MNG: 50, MR: 0.2, CR: 0.5 and KNN: 3.

The SNN parameters were: maximum number of peaks (MNP): automatic detection; upper limit of cycles (ULC): 1000; number of prototypes (NP): automatic detection.

The QC method had only one controllable parameter: the maximum number of peaks (MNP), which was set to automatic detection.

### Methods for cross and external validation

For intra-farm analysis, cross validation of each classifier as performed using 80% of the available spectra for training and the remaining 20% for validation. The procedure was repeated 10 times, each time randomising the extraction of spectra for the training set.

For inter-farm analysis cross validation of the global classifier was performed using 80% of the spectra obtained by aggregation of data from the 19 farms for training and the remaining 20% for validation. The procedure was repeated 10 times, each time randomising the extraction of spectra for the training set. For external validation, 100% of the available aggregated spectra was used for training, and the spectra from the 10 holdout farms were used for validation. The entire procedure was repeated 10 times for the GA classifier, because of the random components present in the method.

### Methods for biomarker characterisation

To find correspondences between the individual peaks of the peak lists and actual proteins of *S. uberis, the* NCBI protein database was used, using a maximum of 0.2% difference in mass as threshold for the successful identification of a correspondence.

The Simple Modular Architecture Research Tool (SMART) was used to find the domains of the proteins. The sequence of hypothetical proteins was pasted into the SMART database search panel. Outlier homologues, as well as homologues of known structure, PFAM domains, signal peptides and internal repeats, were checked. The next step was identifying the homology of these hypothetical proteins in Basic Local Alignment Search Tool (BLASTp). To further investigate the function of the identified proteins (peaks that were cross-referenced with the NCBI *S. uberis* protein database) we studied their protein networks. This was done by analysing the protein-protein interaction (PPI) dataset of *S. uberis*, obtained from STRING database^62^ with Cytoscape 3.6.1^63^. Only genes showing at least two interactions were kept and their functions were detailed along the aspects of biological process, molecular function, cellular component and KEGG pathway. The three dimensional homology models of *S. uberis* were obtained by retrieving the aminoacidic FASTA sequence from Uniprot (https://www.uniprot.org/uniprot/B9DSW3) and using Swiss-Model^64^ to search for templates and build the models. The visualisation shown in Fig. 5 was created using UCSF Chimera^65^.
